# Supplementary material for: Enhancing Insights into Australia’s Gonococcal Surveillance Programme through Stochastic Modelling
Source: Pathogens. 2023 Jul 4;12(7):907. doi: 10.3390/pathogens12070907 (PMC10385950; doi:10.3390/pathogens12070907)
Supplement: Supplementary file 1 [file pathogens-12-00907-s001.zip › Supplementary File - S2 Model Implementation.pdf]

Supplementary File S2 – Model Implementation

R Code with Supplementary Files are available at:
https://github.com/Phu-Do/ScenarioTreeModelling
