# Supplementary material for: Enhancing Insights into Australia’s Gonococcal Surveillance Programme through Stochastic Modelling
Source: Pathogens. 2023 Jul 4;12(7):907. doi: 10.3390/pathogens12070907 (PMC10385950; doi:10.3390/pathogens12070907)

Table S1 Australian Gonococcal Surveillance Program (AGSP) scenario tree Model parameter inputs with respective description, assumptions, and source of information.

Component	Input	Description	Assumptions	Source
$N_{AMenHealth}$	0.52	An estimate parameter for which a male will present to a health service for the possibility the individual will receive a diagnosis and pathology test given they are asymptomatic for N. *gonorrhoeae* infection.	• No direct parameter can be sourced regarding asymptomatic presentation of individuals. Estimate based on annualised proportion of males who visit general practice once.	[1]
$N_{SMenHealth}$	Uniform [0.80-0.99]	An estimate parameter for which a male will present to a health service for the possibility the individual will receive a diagnosis and pathology test given they are symptomatic for N. *gonorrhoeae* infection.	• The parameter is an estimate based on clinician's perceived behaviour regarding probability of presentation. • Uncertainty has been included to account for contextual factors such as accessibility and health literacy which may influence presentation. • Uniform distribution assumes there is equal probability that this proportion of	[2], Expert Opinion[1]

Component	Input	Description	Assumptions	Source
			individuals captures the true population parameter.	
$N_{SWomenHealth}$	Uniform [0.80-0.99]	An estimate parameter for which a female will present to a health service for the possibility the individual will receive a diagnosis and pathology test given they are symptomatic for N. *gonorrhoeae* infection.	• The parameter is an estimate based on clinician's perceived behaviour regarding probability of presentation.<br>• Uncertainty has been included to account for contextual factors such as accessibility and health literacy which may influence presentation.<br>• Uniform distribution assumes there is equal probability that this proportion of individuals captures the true population parameter.	[2], Expert Opinion[1]
$N_{AWomenHealth}$	0.65	An estimate parameter for which a female will present to a health service for the possibility the individual will receive a diagnosis and pathology test given they are asymptomatic for N. *gonorrhoeae* infection.	• Based on the proportion of females attending a general practice clinic over an annualised period.	[1]

Component	Input	Description	Assumptions	Source
**N_{AST}**	Uniform [0.2 – 0.3]	An estimate proportion for the number of N. *gonorrhoeae* isolates that contain associated antibiotic susceptibility data (AST) for the determination of resistance status. The estimate covers isolates that are tested at a laboratory within the National Neisseria Network laboratory for antimicrobial susceptibility. Estimates have been pulled the Australian Gonococcal Surveillance Program reports published within the Communicable Disease Intelligence journal.	• This has been used as a general indicator for clinician initiating susceptibility testing based on evidence of total number of isolates with susceptibility data present.<br>• All tests are results are reported and are subsequently presented in reports.<br>• Does not account for private laboratories.<br>• Same methodology is employed by reference laboratories with comparable results.<br>• The estimate also considers the probability parallel culture has been initiated as evidenced by the proportion of samples with AST data	Expert Opinion[3], [3-5]

Component	Input	Description	Assumptions	Source
$N_{Asymptomatic}$	Uniform [0.05 – 0.15]	The probability that an individual is infected with *N. Gonorrhoeae* and does not display symptoms. Split into sexes due to anatomical differences	• Anatomical differences may influence the recognition of N. *gonorrhoeae* infection and result in different	
$N_{ComHealth}$	Uniform [0.1 – 0.175]	The proportion of tests completed by a community healthcare service. This refers to healthcare services outside of tertiary and general practice primary care such as nurse-led clinics and Indigenous health services.	• Does not take into account resource allocations for community healthcare settings.	Expert Opinion[2]
$N_{ComHealthSym}$	PERT [0.9 – 0.95 – 0.99]	Probability for the initiation of diagnostic testing within a community health setting for both male and female given they are symptomatic	• Uncertainty has been included to do with clinician behaviour, competency, and familiarity with disease	Expert Opinion[2]
$N_{ComHealthAsym}$	Uniform [0.01 – 0.05]	Probability for the initiation of diagnostic testing within a community health setting for both male and female given they are asymptomatic	• Includes follow up locations and maternity care as possible reasons for sexual health screening	Expert Opinion[1]
$N_{ComTest}$	**Symptomatic** PERT	The probability that a test will be initiated by a clinician within a community healthcare setting.	• Assumed to have a similar probability with primary care GPs<br>• Assumes same for males and females	Expert Opinion[1,2]

Component	Input	Description	Assumptions	Source
	[0.9 – 0.95 – 0.99] **Asymptomatic** Uniform [0.01 – 0.05]		• Delay in presentation is still treated as presented (assuming no delay)	
**$N_{FAInitGP}$**	PERT [0.046 – 0.0465 – 0.047]	An estimate probability for which an asymptomatic female attends a general practice clinic and has diagnostic testing initiated by the clinician. This is the precedent event for potential diagnostic and culture events.	• The parameter includes screening for other conditions and/or pregnancy. It assumes this is consistent for detection of *N. Gonorrhoeae* • The assumption is a general check-up is for asymptomatic presentations • Calculated through an average of the points	[6], Expert Opinion[2]
**$N_{SInitGP}$**	PERT [0.925 – 0.9575 – 0.99]	Probability for a general practitioner to initiate diagnostic testing for N. *gonorrhoeae* that is symptomatic	• Includes uncertainty with regards to clinician familiarity, behaviour, health literacy of the patient.	Expert Opinion[1,2]

Component	Input	Description	Assumptions	Source
			• Anatomical differences also introduce some uncertainty.<br>• Assumed to be the same for males and females	
N_{female}		N. *Gonorrhoeae* displays different symptoms based on the anatomical differences. This affects the diagnostic pathways for which an individual presents to get tested.		
$N_{InitSexHealth}$	0.99	The probability a sexual health screening test to detect N. *gonorrhoeae* through a sexual health screening initiated within a sexual health clinic. This includes males and females attending sexual health clinics regardless of symptom status.	• The parameter includes follow up and initial consultation at a sexual health clinic.<br>• Sexual health clinics are assumed for individuals to already want to have sexual health testing completed and will be mandatory.<br>• The probability for males and females is the same regardless of symptom status	Expert Opinion[1]

Component	Input	Description	Assumptions	Source
$N_{FSInitGP}$	PERT [0.70 – 0.75 – 0.80]	The probability that a diagnosis is initiated completed within the primary health context with a general practitioner when an individual is symptomatic. The parameter is inclusive of public and private healthcare. Uncertainty has been included to factor in determinants such as competency and familiarity with disease have been included within the parameter. The parameter is inclusive of symptomatic males and females.	• It is assumed with an individual is symptomatic there is a high likelihood for the clinician to initiate diagnostic testing.<br>• Uncertainty for correct tests due to familiarity with condition has been factored into the parameter	
$N_{MAInitGP}$	PERT[0.04 – 0.0475 – 0.055]	The probability an asymptomatic male undergoes a general check-up as indicated by pathology requests to initiate diagnosis.	• The parameter includes screening for other conditions. It assumes this is consistent for detection of *N. Gonorrhoeae*<br>• The assumption is a general check-up is for asymptomatic presentations<br>• Calculated through an average of the minimum and maximum	[6]

Component	Input	Description	Assumptions	Source
N_{male}		*N. Gonorrhoeae* displays different symptoms based on the anatomical differences. This affects the diagnostic pathways for which an individual presents to get tested.		
$N_{MenAsymptom}$	Uniform[0.05 – 0.10]	The proportion of men that are asymptomatic with *N. Gonorrhoeae*	• The complement of $N_{MenSymptom}$	[7]
$N_{MenSymptom}$	Uniform[0.85 – 0.90]	The proportion of men that are symptomatic with *N. Gonorrhoeae*	• Symptomatic with urogenital, anorectal, or pharyngeal symptoms	[7]
NSe_{Cult}	Uniform[0.85 – 0.99]	The sensitivity of the culture to determine antibiotic susceptibility for the *N. Gonorrhoeae* isolate.	• The parameter for assumes a usable sample will be presented for culture.<br>• Assumes the initiation of parallel culture or culture during follow up event with N. *gonorrhoeae* infection.	[8]
NSe_{Test}	Uniform[0.85 – 1]	The sensitivity of the diagnostic performed to diagnose *N. Gonorrhoeae*.	• Testing sensitivity is dependent test type. The parameter is a pooled estimate of Microscopy, and NAAT.	[8]

Component	Input	Description	Assumptions	Source
$N_{SexHealth}$	Uniform [0.1 – 0.175]	The proportion of tests completed at a sexual health clinic. The clinics are dedicated to sexually transmittable disease testing.	• Remains constant and does not change across strata.	Expert Opinion[2]
N_{GP}	PERT [0.70 – 0.75 – 0.80]	The proportion of tests completed by general practitioners	• There is an assumption that primary healthcare services write the most pathology requests. • There are negligible differences between the intensity of testing between geographical locations as it is difficult to parameterise.	[6], Expert Opinion[1]
$N_{SInitGP}$	PERT [0.90 – 0.95 – 0.99]	The probability that a diagnosis is initiated completed within the primary health context with a general practitioner when an individual is symptomatic. The parameter is inclusive of public and private healthcare. Uncertainty of clinician competency has	• It is assumed with an individual is symptomatic there is a high likelihood for the clinician to initiate diagnostic testing	Expert Opinion[1]

Component	Input	Description	Assumptions	Source
		been included within the parameter. The parameter is inclusive of males and females.	• Uncertainty for correct tests due to familiarity with condition has been factored into the parameter	
$N_{Tertiary}$	Uniform[0.01 – 0.05]	The proportion of sexual tests completed within a tertiary healthcare setting. Excluding those within a collected within an outpatient setting.	• Assumed to be the same for asymptomatic and symptomatic presentations<br>• For women pregnant women testing is assumed to be much greater at antenatal visits and thus probability for testing sequentially is affected	Expert Opinion[1,2]
$N_{AInitTertiary}$	**Male**<br>Uniform [0.01 – 0.05]<br>**Female**<br>Uniform [0.02 – 0.06]	An estimate probability of having diagnostic testing initiated for those asymptomatic within a tertiary healthcare setting. Tertiary care includes routine hospital inpatient as well as emergency department.	• Antenatal care stipulates routine sexual health testing is a core component.<br>• Sexual health testing in tertiary care for males is uncommon	Expert Opinion[1]

Component	Input	Description	Assumptions	Source
**$N_{SInitTertiary}$**	PERT [0.90 – 0.95 – 0.99]	Estimate probability of the initiation of diagnostic testing for those symptomatic within a tertiary healthcare setting.	• Tertiary healthcare setting will indicate no resource constraints and will allow for immediate testing with full capacity.<br>• Does not consider different varying resource constraints of the hospital.	Expert Opinion[1,2]
**$N_{WomenAsymptom}$**	0.8	The proportion of women who are asymptomatic with *N. Gonorrhoeae*	• Complement of $N_{WomenSymptom}$	[7]
**$N_{WomenSymptom}$**	0.2	The proportion of women that are symptomatic with *N. Gonorrhoeae*	• Symptomatic with urogenital, anorectal, or pharyngeal symptoms	[7]

[1] Sexual health nurse academic in public health

[2] Infectious disease physician

[3] Clinical microbiologist involved in the Australian Gonococcal Surveillance Program (AGSP)

References

1. ABS: **Patient Experiences in Australia: Summary of Findings**. Australian Bureau of Statistics [https://www.abs.gov.au/statistics/health/health-services/patient-experiences-australia-summary-findings/latest-release#data-download]
2. Patel CG, Tao G: **STI/HIV Testing and Prevalence of Gonorrhea and Chlamydia Among Persons with Their Specified-Type Sex Partner**. *Am J Med* 2022, **135**(2):196-201.
3. Lahra MM, Hogan TR, Shoushtari M, Armstrong BH: **Australian Gonococcal Surveillance Programme Annual Report, 2020**. *Commun Dis Intell (2018)* 2021, **45**.
4. Lahra MM, Hogan TR: **Australian Gonococcal Surveillance Programme Annual Report, 2021**. *Communicable Diseases Intelligence* 2022, **46**.
5. Trembizki E, Jennison AV, Buckley C, Bright A, Holds J, Ward A, Pitt J, Pendle S, Baird R, Freeman K, Robson J, Mhango L, Lowry K, Lahra M, Whiley D: **Enhanced molecular surveillance in response to the detection of extensively resistant gonorrhoea in Australia**. *J Antimicrob Chemother* 2021, **76**(1):270-271.
6. C. Bayram, L. Venti: **GP pathology ordering**. *General practice in Australia: health priorities and policies 1998 to 2008 General practice series no 24 Cat no GEP 24* 2009.
7. ASHM: **Gonorrhoea** [https://sti.guidelines.org.au/sexually-transmissible-infections/gonorrhoea/]
8. PHLN: **Gonococcal infection (Neisseria gonorrhoeae): Laboratory case definition.** Public Health Laboratory Network; 2002 [https://www.health.gov.au/sites/default/files/documents/2022/06/gonococcal-infection-laboratory-case-definition_0.pdf]

Table 2 Gonococcal component sensitivities

sex	symptom	location	mean	ower95	upper95
men	asymptom	community	0.00013227084	0.00013183855	0.00013270313
men	asymptom	gp	0.00114149192	0.00113934414	0.00114363969
men	asymptom	sexhealth	0.00435833223	0.00434918102	0.00436748344
men	asymptom	tertiary	0.00002882314	0.00002870564	0.00002894064
men	symptom	community	0.06506986705	0.06499436214	0.06514537196
men	symptom	gp	0.35824206798	0.35801651846	0.35846761751
men	symptom	sexhealth	0.06785288932	0.06777487052	0.06793090811
men	symptom	tertiary	0.01419671074	0.01416161979	0.01423180170
women	asymptom	community	0.00068616446	0.00068472689	0.00068760202
women	asymptom	gp	0.00434404361	0.00434213616	0.00434595107
women	asymptom	sexhealth	0.01694247876	0.01692454907	0.01696040845
women	asymptom	tertiary	0.00014954539	0.00014908257	0.00015000821
women	symptom	community	0.00562475060	0.00561832117	0.00563118002
women	symptom	gp	0.03096334928	0.03094486804	0.03098183052
women	symptom	sexhealth	0.00586493291	0.00585828420	0.00587158163
women	symptom	tertiary	0.00122705249	0.00122403173	0.00123007325

Table 3 Antibiotic resistance component sensitivities

sex	symptom	location	mean	lower95	upper95
men	asymptom	community	0.000033069132	0.000032957919	0.00003318035
men	asymptom	gp	0.000285379918	0.000284802544	0.00028595729
men	asymptom	sexhealth	0.001089702140	0.001087270458	0.00109213382
men	asymptom	tertiary	0.000007205352	0.000007175363	0.00000723534
men	symptom	community	0.016272204663	0.016249845770	0.01629456356
men	symptom	gp	0.089576155660	0.089490280164	0.08966203116
men	symptom	sexhealth	0.016967594075	0.016944430819	0.01699075733
men	symptom	tertiary	0.003549775995	0.003540581836	0.00355897015
women	asymptom	community	0.000171558696	0.000171176927	0.00017194046
women	asymptom	gp	0.001086139213	0.001085224980	0.00108705345
women	asymptom	sexhealth	0.004236483691	0.004231034949	0.00424193243
women	asymptom	tertiary	0.000037390074	0.000037270568	0.00003750958
women	symptom	community	0.001406531618	0.001404621325	0.00140844191
women	symptom	gp	0.007741778963	0.007734539716	0.00774901821
women	symptom	sexhealth	0.001466535552	0.001464556251	0.00146851485
women	symptom	tertiary	0.000306792255	0.000306001226	0.00030758328

Figure 1 Full scenario tree model with parameters of the Australian gonococcal surveillance system program (AGSP)

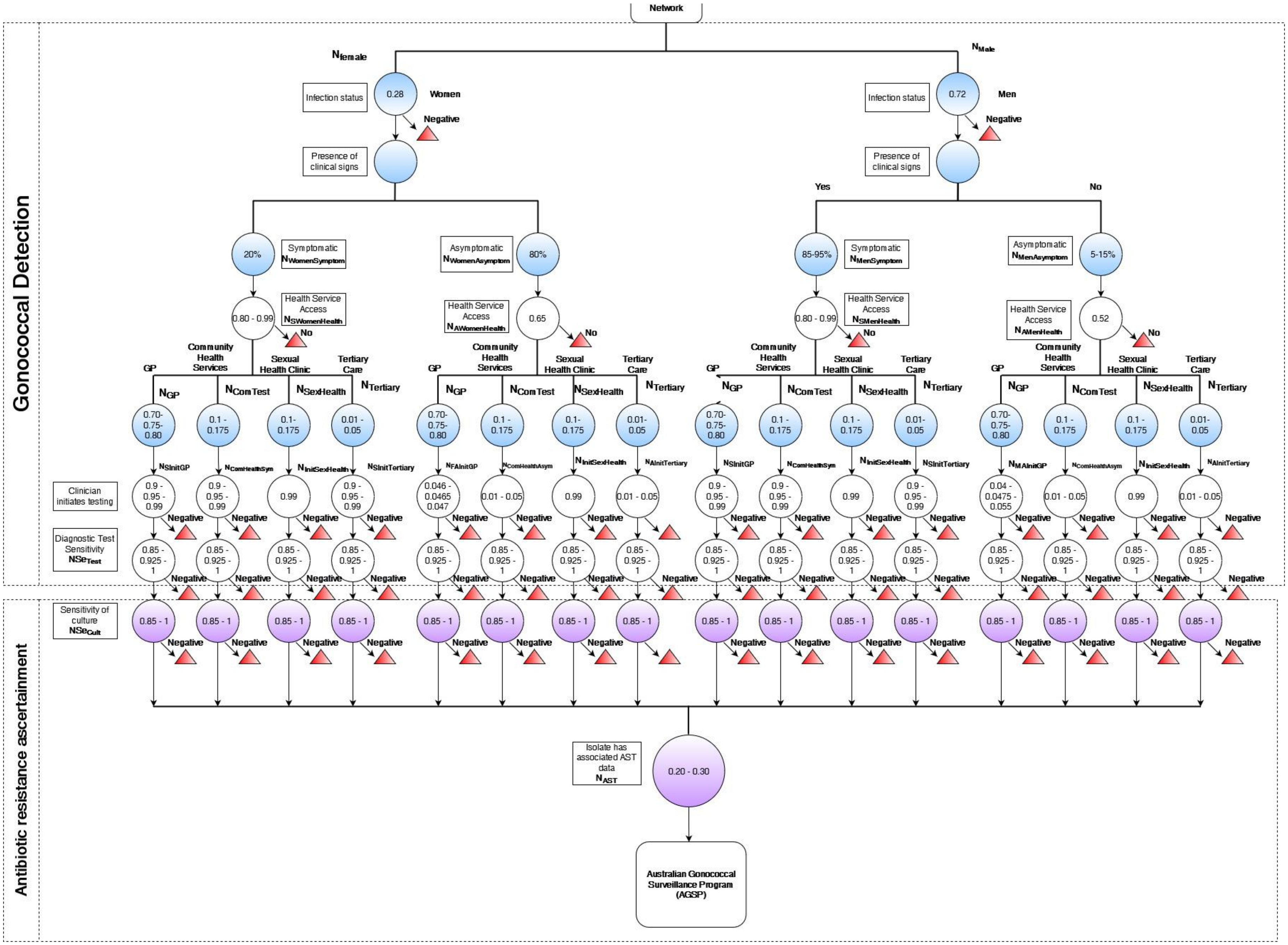

Supplement: Supplementary file 1 [file pathogens-12-00907-s001.zip › Supplementary File S1 - Model inputs parameters and outputs.pdf]
